# Skeletal muscle index based on CT at the 12th thoracic spine level can predict osteoporosis and fracture risk: a propensity score-matched cohort study

**DOI:** 10.3389/fmed.2024.1387807

**Published:** 2024-04-25

**Authors:** Jia-sen Hu, Ya-ping Jin, Ji-kui Wu, Jian-guang Ni

**Affiliations:** Department of Orthopedics, Affiliated Yueqing Hospital of Wenzhou Medical University, Wenzhou, China

**Keywords:** osteoporosis, skeletal muscle index, fracture risk assessment tool, propensity score matching, computed tomography, bone mineral density

## Abstract

**Background:**

Multiple studies have shown that skeletal muscle index (SMI) measured on abdominal computed tomography (CT) is strongly associated with bone mineral density (BMD) and fracture risk as estimated by the fracture risk assessment tool (FRAX). Although some studies have reported that SMI at the level of the 12th thoracic vertebra (T12) measured on chest CT images can be used to diagnose sarcopenia, it is regrettable that no studies have investigated the relationship between SMI at T12 level and BMD or fracture risk. Therefore, we further investigated the relationship between SMI at T12 level and FRAX-estimated BMD and fracture risk in this study.

**Methods:**

A total of 349 subjects were included in this study. After 1∶1 propensity score matching (PSM) on height, weight, hypertension, diabetes, hyperlipidemia, hyperuricemia, body mass index (BMI), age, and gender, 162 subjects were finally included. The SMI, BMD, and FRAX score of the 162 participants were obtained. The correlation between SMI and BMD, as well as SMI and FRAX, was assessed using Spearman rank correlation. Additionally, the effectiveness of each index in predicting osteoporosis was evaluated through the receiver operating characteristic (ROC) curve analysis.

**Results:**

The BMD of the lumbar spine (L1-4) demonstrated a strong correlation with SMI (*r* = 0.416, *p* < 0.001), while the BMD of the femoral neck (FN) also exhibited a correlation with SMI (*r* = 0.307, *p* < 0.001). SMI was significantly correlated with FRAX, both without and with BMD at the FN, for major osteoporotic fractures (*r* = −0.416, *p* < 0.001, and *r* = −0.431, *p* < 0.001, respectively) and hip fractures (*r* = −0.357, *p* < 0.001, and *r* = −0.311, *p* < 0.001, respectively). Moreover, the SMI of the non-osteoporosis group was significantly higher than that of the osteoporosis group (*p* < 0.001). SMI effectively predicts osteoporosis, with an area under the curve of 0.834 (95% confidence interval 0.771–0.897, *p* < 0.001).

**Conclusion:**

SMI based on CT images of the 12th thoracic vertebrae can effectively diagnose osteoporosis and predict fracture risk. Therefore, SMI can make secondary use of chest CT to screen people who are prone to osteoporosis and fracture, and carry out timely medical intervention.

## Introduction

The loss of bone mass and skeletal muscle is often one of the important triggers of falls and prolonged bed rest in the elderly population (1, 2). Studies have shown that the body loses muscle mass at a rate of 1% per year by the age of 40 and older (3). Sarcopenia is an age-related decline in overall muscle mass and strength or physical muscle function—a degenerative disease resulting in a marked decrease in balance and mobility (4). The primary diagnostic approach for sarcopenia in clinical guidelines involves assessing participants’ skeletal muscle index (SMI) using methods like dual-energy X-ray absorptiometry (DXA) and bioelectrical impedance analysis (BIA) (5). In addition, many studies have shown that SMI can be well reflected by calculating the area of a single chest or abdominal CT image (6–8). Shen et al. (6) demonstrated that SMI based on T12 levels can predict in-hospital mortality in elderly patients. In addition, Tan et al. (7) demonstrated that SMI of the T12 thoracic vertebrae contributes to the diagnosis of sarcopenia in the Chinese population. Based on the above studies, SMI at the T12 level may be a potential indicator to predict osteoporosis and fracture risk propensity.

Osteoporosis is a bone metabolic disorder in which the loss of bone mass causes changes in the microstructure of bone, resulting in decreased mechanical structural properties and increased fragility of bone (9, 10). Risk factors for osteoporosis include aging, sex (more common in women), family genetics, poor diet, lack of exercise, and certain chronic diseases (11). Similarly, these risk factors are strongly associated with changes in muscle mass (12). Bone mineral density (BMD) assessment via DXA serves as the gold standard for diagnosing osteoporosis (13). The Fracture risk assessment tool (FRAX) is often applied to predict the risk of osteoporotic fracture in individuals, such as a femoral neck (FN) fracture in the elderly, using risk factors associated with osteoporosis (14, 15). In addition, FRAX is widely used worldwide because of the reliability and ease of use of its predictive results.

There have been several studies showing that osteoporosis is strongly associated with sarcopenia (16, 17). Multiple studies have shown that SMI measured on abdominal computed tomography (CT) is strongly associated with BMD and fracture risk (18, 19). Although the authors demonstrated that SMI measured on CT images at the level of the 12th thoracic vertebra (T12) can be used to diagnose sarcopenia (7), no subsequent studies have delved into the correlation between SMI and BMD at the T12 level, or the connection between SMI and fracture risk. In addition, chest CT has been more and more widely used in physical examination and hospitalization, such as follow-up of lung nodules and screening of lung cancer.

In summary, in this study, our team explored the relationship between SMI and BMD at the T12 level and the relationship between SMI and fracture risk. When patients undergo a chest CT examination (for other clinical reasons), clinicians have the opportunity to use these imaging data to screen for osteoporosis and fracture risk.

## Methods

### Study participants

With the approval of the review board of Affiliated Yueqing Hospital of Wenzhou Medical University, patients who completed DXA and chest CT examinations in the database of the Yueqing Hospital of Wenzhou Medical University from January 1, 2023 to December 1, 2023 were retrospectively collected. The inclusion criteria for this study were (1) Unenhanced chest CT scan was performed, (2) DXA was performed, and (3) Complete medical records. The exclusion criteria for this study were (1) The interval between DXA and chest CT examination was >3 months, (2) Artifacts on CT images, and (3) Lack of medical records. A total of 349 subjects were enrolled in this study, and 162 of them were finally included after propensity score matching (PSM) analysis. Table 1 presents the demographic and clinical baseline characteristics of the study participants.

**Table 1:** Comparison of clinical characteristics between two groups: before and after the propensity matching score.

	Overall series			Propensity score–matched pairs		
	Non-osteoporosis (249)	Osteoporosis(100)	*P*-value	Non-osteoporosis (81)	Osteoporosis(81)	*P*-value
Age (years)	63(57–71)	67(63–74)	<0.001	64(59–72)	68(63–74)	0.028
Height (cm)	161(156–168)	156(153–161)	<0.001	157(153–162)	156(153–161)	0.672
Weight (kg)	63.60 ± 8.96	57.19 ± 9.31	<0.001	60.23 ± 8.86	59.26 ± 8.58	0.153
BMI (kg/m^2^)	24.29 ± 3.58	23.17 ± 3.63	0.010	24.12 ± 3.53	23.58 ± 3.47	0.320
Gender			<0.001			0.693
Female, n(%)	137(55.0)	84(84.0)		64(79.0)	66(81.5)	
Male, n(%)	112(45.0)	16(16.0)		17(21.0)	15(18.5)	
Hypertension, n(%)	159(63.9)	59(59.0)	0.397	47(58.0)	51(63.0)	0.520
Diabetes, n(%)	238(97.9)	84(92.3)	0.033	79(97.5)	78(96.3)	1.000
Hyperlipidemia, n(%)	147(59.0)	56(56.0)	0.603	51(63.0)	48(59.3)	0.629
Hyperuricemia, n(%)	30(12.0)	7(7.0)	0.166	5(6.2)	5(6.2)	1.000

BMI, body mass index.

### SMI measurements

**Figure 1:**
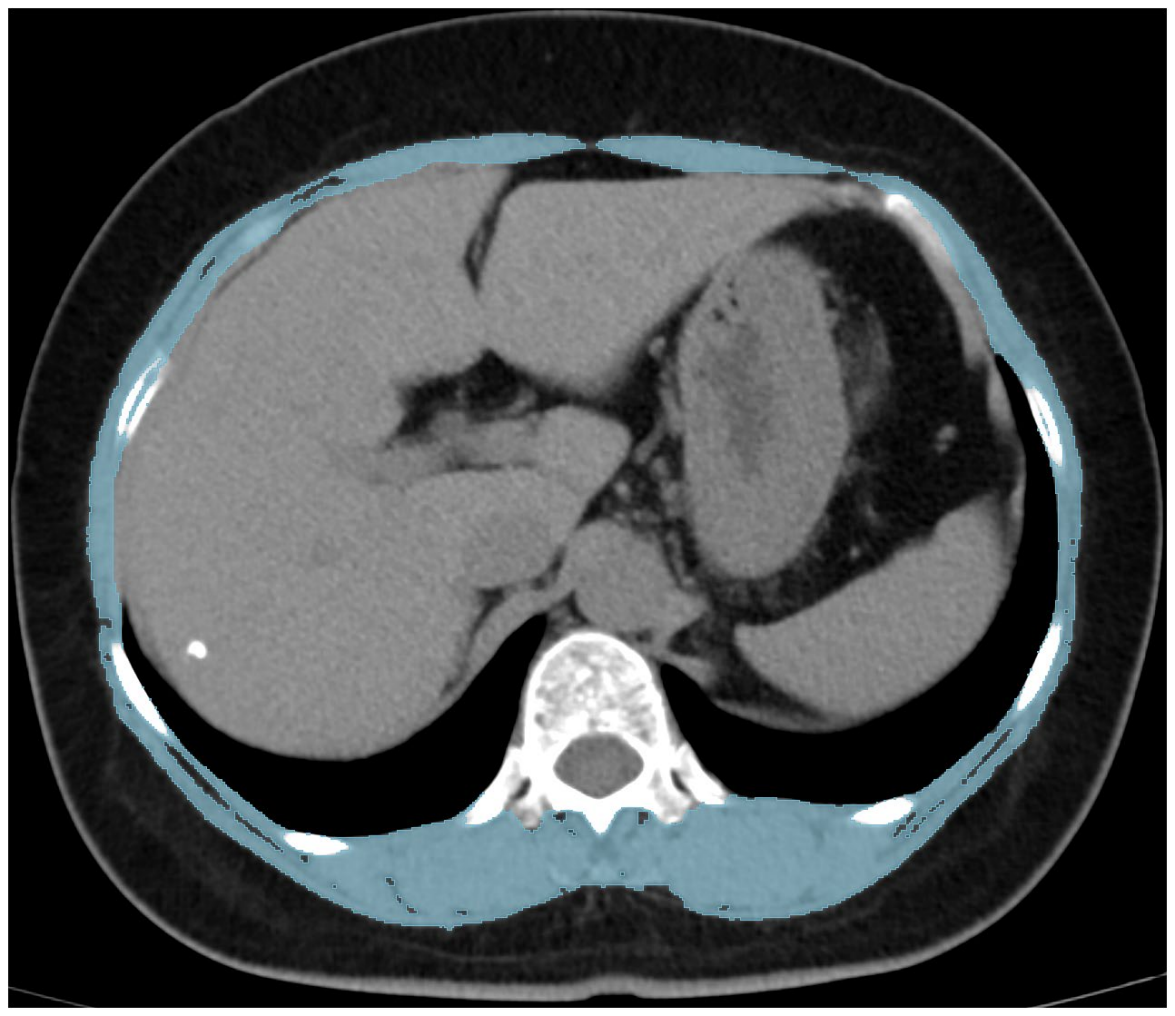
Measurement of the skeletal muscle index using computed tomography at the T12 level. T12 the 12th thoracic vertebra.

A 120 kV, 250 mA, 5 mm slice thickness picture archiving and communication system was used to collect CT image data. Moreover, the CT images were obtained within 3 months after DXA examination to keep bone mass and muscle mass data in the same period as possible. Skeletal muscle cross-sectional area at the middle level of the T12 vertebral body was calculated using Image J (NIH Image J version 1.52c) software (Figure 1). Relevant studies (20) have shown that the skeletal muscle threshold ranges from −29HU to 150HU, and the skeletal muscle area was measured within this threshold range in this study. SMI was derived by dividing the calculated area value by the square of the patient’s height (m^2^).

### BMD and diagnosis of osteoporosis

BMD at the entire lumbar (lumbar spine 1–4, L1-4) and FN were measured by DXA. According to relevant clinical guidelines, patients with osteoporosis are diagnosed based on the T or Z scores of the femoral neck and lumbar spine measured by DXA.

### The FRAX tool

A questionnaire survey on the risk factors of fracture was conducted by face to face or telephone. History of fractures, secondary osteoporosis, glucocorticoid use, parents with hip fractures, excess alcohol intake, gender, current smoking, age, systemic rheumatoid arthritis, height, weight, and FN of BMD were included. Through the https://www.sheffield.ac.uk/FRAX/?lang=chs website login Chinese version FRAX model, and the clinical data of patients after PSM is applied to the model. The 10-year probability of osteoporotic fracture was calculated on whether to include FN BMD in the FRAX model.

### Statistics

The Shapiro–Wilk test assessed data distribution. Subject baseline characteristics were described using medians (interquartile range), means ± standard deviations, frequencies, and percentages. Nonparametric tests were employed for non-normally distributed or heteroscedastic data. Categorical variables were analyzed using the Pearson Chi-squared test. Spearman rank correlation was used to assess the correlation between SMI and BMD, as well as SMI and FRAX. The receiver operating characteristic curve (ROC) was utilized to evaluate the predictive efficacy of each index for osteoporosis. Statistical analyses were conducted using SPSS software (version 26.0; SPSS Inc., Chicago, IL, United States).

Propensity Score Matching (PSM) is frequently employed in observational studies to address covariate imbalances between groups. It involves employing specific statistical methods to screen the experimental and control groups, facilitating a more equitable comparison between them (21). In addition, some studies have shown that the results of PSM analysis are close to those of prospective random cohort studies (22). Nine covariates were included in this study, including age, height, hypertension, weight, diabetes, body mass index (BMI), hyperlipidemia, gender, and hyperuricemia. On the basis of these covariates, using propensity scores based on logistic regression, the nearest neighbor matching was applied to generate pairs of subjects in the osteoporosis and non-osteoporosis groups. A preset caliper width of 0.1 was used and no cases were replaced. PSM was calculated using Statistical Package for Social Science software version 26.0 (SPSS, Chicago, Illinois, United States).

## Results

Table 1 displays clinical characteristics data for the eligible patients. This study included 349 subjects, with 249 in the non-osteoporosis group and 100 in the osteoporosis group. Significant differences were observed between the two groups in terms of age, gender, height, weight, BMI, and diabetes (*p*-values were all <0.05). However, there were no significant differences in hypertension, hyperlipidemia, and hyperuricemia between the two groups. After PSM analysis, 162 subjects were enrolled in the study, including 81 in the non-osteoporosis group and 81 in the osteoporosis group. Furthermore, age was the only significant difference between the two groups (64 versus 68 *p* = 0.028), while height, weight, BMI, sex, hypertension, hyperlipidemia, diabetes, and hyperuricemia were not. Patients obtained from PSM analysis were evaluated for fracture risk, and their fracture risk factors are shown in Table 2. Among the 162 patients, 22 had a history of brittle fractures, 18 had a history of smoking, 3 had a history of parental hip fractures, 4 had a history of systemic glucocorticoid use, 4 had a history of rheumatoid arthritis, 27 had a history of risk factors for secondary osteoporosis, and 13 had a history of excess alcohol intake.

**Table 2:** Prevalence of factors associated with the FRAX after propensity score matching analysis

Fracture-related factor	n (%)
Prior fragility fracture	22(13.6)
Parental hip fracture	3(1.9)
Smoking	18(11.1)
Systemic glucocorticoid use	4(2.5)
Rheumatoid arthritis	4(2.5)
Other cases of secondary osteoporosis	27(16.7)
Excess alcohol intake	13(8.0)

FRAX, Fracture Risk Assessment Tool.

The measurement results after propensity score matching analysis are presented in Table 3. The SMI was 32.46 ± 6.64 cm^2^/m^2^. L1-4 and FN BMD were 0.91 ± 0.13 g/cm^2^ and 0.74 ± 0.11 g/cm^2^, respectively. Moreover, the probability of major osteoporotic fractures was 6.59 ± 5.14% and 2.33 ± 3.55% for hip fractures when the femoral neck BMD was included. The probability of major osteoporotic fractures was 6.63 ± 4.11% and 2.48 ± 3.61% for hip fractures when the femoral neck BMD was not included.

**Table 3:** The measurement results after propensity score matching analysis

Characteristic	Value
SMI (cm^2^/m^2^)	32.46 ± 6.64
BMD (g/cm^2^)	
L1-L4	0.91 ± 0.13
Femoral neck	0.74 ± 0.11
**FRAX (10-year probability of fracture), %**	
Major osteoporotic fracture (with BMD at femoral neck)	6.59 ± 5.14
Hip fracture (with BMD at femoral neck)	2.33 ± 3.55
Major osteoporotic fracture (without BMD at femoral neck)	6.63 ± 4.11
Hip fracture (without BMD at femoral neck)	2.48 ± 3.61

BMD, bone mineral density; SMI, skeletal muscle index; FRAX, Fracture Risk Assessment Tool.

**Figure 2:**
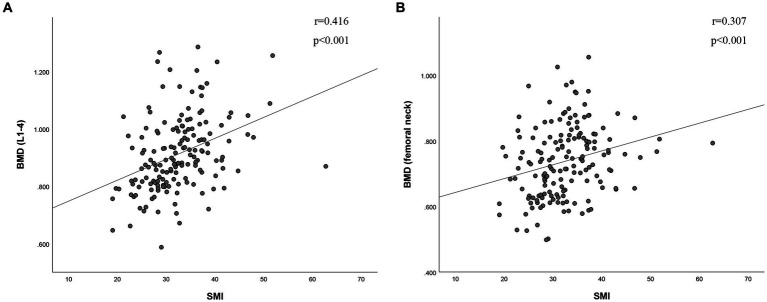
The correlation between skeletal muscle index and **(A)** bone mineral density of L1-4 and **(B)** bone mineral density of femoral neck. BMD (g/cm^2^), bone mineral density; SMI (cm^2^/m^2^), skeletal muscle index; L1-4, lumbar spine 1–4.

**Figure 3:**
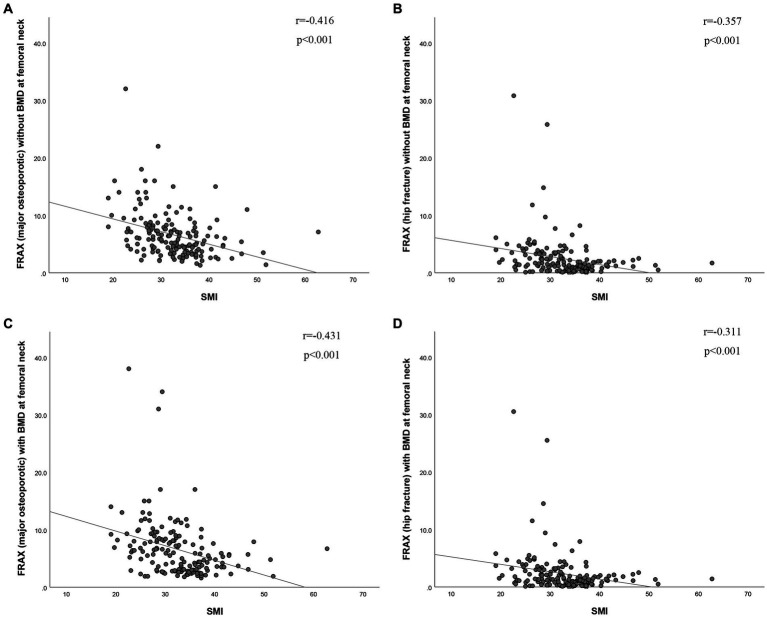
The correlation between skeletal muscle index and risk of **(A,C)** major osteoporotic fractures and **(B,D)** hip fractures (with or without BMD of the femoral neck). BMD (g/cm^2^), bone mineral density; SMI (cm^2^/m^2^), skeletal muscle index; FRAX, fracture risk assessment tool.

**Figure 4:**
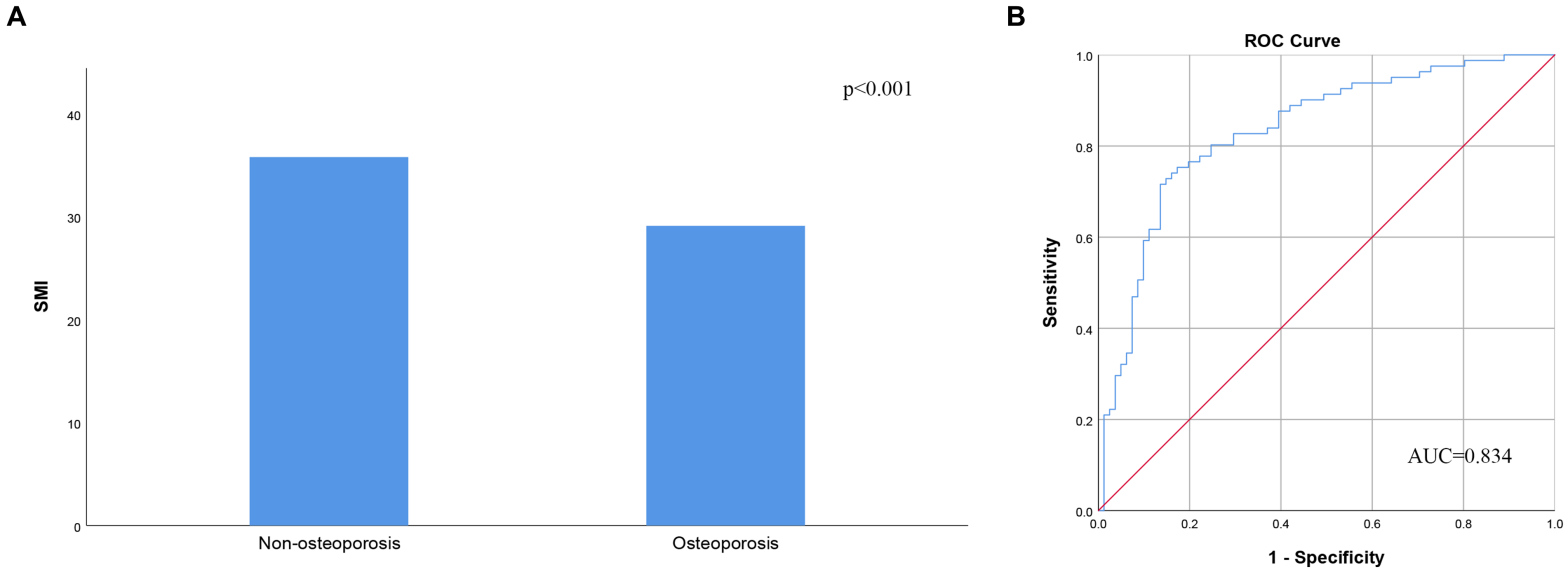
**(A)** Skeletal muscle index according to the presence of osteoporosis. **(B)** Receiver operating characteristic analysis of skeletal muscle index’s ability to diagnose osteoporosis. AUC, area under the curve; SMI, skeletal muscle index.

The BMD of L1-4 exhibited a strong correlation with SMI (*r* = 0.416, *p* < 0.001, as shown in Figure 2A). Additionally, BMD of the FN showed a correlation with SMI (*r* = 0.307, *p* < 0.001, as depicted in Figure 2B). SMI also displayed correlations with FRAX, both with and without BMD at the FN. For major osteoporotic fractures, these correlations were as follows: *r* = −0.431, *p* < 0.0001, and *r* = −0.416, *p* < 0.001, respectively. For hip fractures, the correlations were: *r* = −0.311, *p* < 0.001, and *r* = −0.357, *p* < 0.001 (refer to Figure 3). Furthermore, Figure 4A illustrates that the SMI of the non-osteoporosis group was significantly higher than that of the osteoporosis group (*p* < 0.001). SMI proved to be an effective predictor of osteoporosis, with an area under the curve of 0.834 (95% confidence interval 0.771–0.897, *p* < 0.001). In order to visualize the prediction performance of SMI, the ROC curve was drawn, as shown in Figure 4B.

## Discussion

Our findings indicate that SMI, measured at the T12 level, demonstrates a robust correlation with both lumbar spine and femoral neck (FN) BMD. Moreover, SMI serves as a reliable predictor of osteoporosis. Additionally, our results show a strong association between SMI and fracture risk as calculated by FRAX.

Osteoporosis is closely related to skeletal muscle; the two interact to make older people prone to falls and brittle fractures. Reduced muscle mass, strength, and function increase the risk of osteoporosis, and similarly, reduced bone mass increases the risk of sarcopenia (23). The bone and skeletal muscle relationship is not just a simple mechanical one. Bone and muscle are endocrine target organs and secretory organs interacting with each other through paracrine and endocrine signals. The skeletal muscle is the largest endocrine organ in the human body, secreting factors that regulate bone metabolism. These muscle-secreting factors mainly include myostatin, β-aminoisobutyric acid and irisin, and so on. In addition, circadian rhythm s, nutritional deficiencies, aging, and nervous system networks also affect bone and muscle (24–26). Therefore, maintaining skeletal muscle mass can prevent sarcopenia and osteoporosis. In this study, SMI based on T12 was closely related to BMD of the FN and lumbar spine. Similar to previous studies, SMI measured by DXA or BIA was strongly associated with BMD (27, 28). In addition, we found that low-quality SMI was effective in predicting osteoporosis, with moderate predictive power after ROC analysis. These results further support the close relationship between SMI and BMD.

Due to the lack of awareness of osteoporosis in the general population, the first diagnosis of osteoporosis is usually found at the time of fracture using DXA examination. However, when patients develop osteoporotic fractures, they have a poorer prognosis and higher mortality than non-osteoporotic fractures (29, 30). Therefore, more and more research is devoted to using a more convenient method to detect osteoporosis, treat osteoporosis in time and prevent osteoporosis fractures. Among them, Kajiki et al. (18) proved that psoas muscle index, as measured by abdominal CT, is strongly associated with the BMD and is a valuable predictor of osteoporosis. However, most patients do not have a specific reason to undergo abdominal CT. Chest CT has been more and more widely used, especially for the follow-up of lung nodules and screening of lung cancer in middle-aged and elderly people. This study found that SMI based on chest CT measurements can predict osteoporosis, providing a new way to more easily screen for osteoporosis. Therefore, when patients undergo chest CT examination (for other clinical reasons), doctors have the opportunity to use these image data for osteoporosis screening, thereby reducing the waste of medical resources to a certain extent.

In this study, SMI exhibited a strong association with fracture risk, as evaluated using FRAX. This outcome aligns with previous research findings that demonstrated a higher fracture risk assessed by FRAX in individuals with low muscle mass (31–33). FRAX effectively improves patient lifestyles and prevents fractures, reducing fracture-related mortality (34). Our study suggests that SMI can be used as an indicator to assess a patient’s risk of fracture. SMI based on chest CT measurement is helpful for early detection of high-risk patients and timely intervention.

This study found that SMI based on chest CT measurements can be used to predict osteoporosis and fracture risk. With the development of medical technology, chest CT has been widely used, especially in routine physical examination. With clinicians’ secondary use of these chest CT images to detect osteoporosis early through SMI, patients can benefit from timely intervention and improved management, potentially reducing the risk of fractures and other related complications. Patients can both improve their quality of life and reduce subsequent medical costs.

### Limitations

This study mainly has the following limitations. First of all, the study is a retrospective study, so there may be a series of selection and recall biases. However, this study used PSM analysis, which can effectively reduce these biases and may even approximate the results of prospective studies. Secondly, the PSM analysis method was used, which resulted in a small number of participants in this study. Therefore, large-scale prospective cohort studies are needed to further support this study. Third, although the FRAX score was used in this study as a predictor of osteoporotic fractures, the relationship between SMI and actual osteoporotic fractures has not been elucidated. Therefore, relevant research is urgently needed in the future.

## Conclusion

SMI based on CT images of the 12th thoracic vertebrae can effectively diagnose osteoporosis and predict fracture risk. Therefore, SMI can make secondary use of chest CT images to screen people who are prone to osteoporosis and fracture, and carry out timely medical intervention to reduce the waste of medical resources to a certain extent.

## Data availability statement

The raw data supporting the conclusions of this article will be made available by the authors, without undue reservation.

## Ethics statement

The studies involving humans were approved by the Department of Orthopedics, Affiliated Yueqing Hospital of Wenzhou Medical University, Wenzhou, China. The studies were conducted in accordance with the local legislation and institutional requirements. Written informed consent for participation was not required from the participants or the participants’ legal guardians/next of kin because this study is a retrospective study and does not involve any impact on patient health and safety.

## Author contributions

J-sH: Data curation, Formal analysis, Writing – original draft. Y-pJ: Data curation, Writing – original draft. J-kW: Data curation, Writing – original draft. J-gN: Writing – review & editing.

## References

[1] Daly RM Dalla Via J Duckham RL Fraser SF Helge EW. Exercise for the prevention of osteoporosis in postmenopausal women: an evidence-based guide to the optimal prescription. Braz J Phys Ther. (2019) 23:170–80. doi: 10.1016/j.bjpt.2018.11.011. PMID:30503353, PMID: 30503353 PMC6429007

[2] Granacher U Gollhofer A Hortobágyi T Kressig RW Muehlbauer T. The importance of trunk muscle strength for balance, functional performance, and fall prevention in seniors: a systematic review. Sports Med. (2013) 43:627–41. doi: 10.1007/s40279-013-0041-1. PMID:23568373, PMID: 23568373

[3] Janssen I Heymsfield SB Wang ZM Ross R. Skeletal muscle mass and distribution in 468 men and women aged 18-88 yr. J Appl Physiol. (2000) 89:81–8. doi: 10.1152/jappl.2000.89.1.81, PMID: 10904038

[4] Papadopoulou SK. Sarcopenia: a contemporary health problem among older adult populations. Nutrients. (2020) 12:1293. doi: 10.3390/nu12051293. PMID:32370051, PMID: 32370051 PMC7282252

[5] Chen LK Woo J Assantachai P Auyeung TW Chou MY Iijima K. Asian working group for Sarcopenia: 2019 consensus update on sarcopenia diagnosis and treatment. J Am Med Dir Assoc. (2020) 21:300–307.e2. doi: 10.1016/j.jamda.2019.12.012. PMID:32033882, PMID: 32033882

[6] Shen Y Luo L Fu H Xie L Zhang W Lu J. Chest computed tomography-derived muscle mass and quality indicators, in-hospital outcomes, and costs in older inpatients. J Cachexia Sarcopenia Muscle. (2022) 13:966–75. doi: 10.1002/jcsm.12948. PMID:35178898, PMID: 35178898 PMC8977961

[7] Tan L Ji G Bao T Fu H Yang L Yang M. Diagnosing sarcopenia and myosteatosis based on chest computed tomography images in healthy Chinese adults. Insights Imaging. (2021) 12:163. doi: 10.1186/s13244-021-01106-2. PMID:34743259, PMID: 34743259 PMC8572237

[8] Shen W Punyanitya M Wang Z Gallagher D St-Onge MP Albu J. Total body skeletal muscle and adipose tissue volumes: estimation from a single abdominal cross-sectional image. J Appl Physiol (1985). (2004) 97:2333–8. doi: 10.1152/japplphysiol.00744.2004, PMID: 15310748

[9] Johnston CB Dagar M. Osteoporosis in older adults. Med Clin North Am. (2020) 104:873–84. doi: 10.1016/j.mcna.2020.06.004 PMID:32773051 32773051

[10] Arceo-Mendoza RM Camacho PM. Postmenopausal osteoporosis: latest guidelines. Endocrinol Metab Clin N Am. (2021) 50:167–78. doi: 10.1016/j.ecl.2021.03.009, PMID: 34023036

[11] Xiao PL Cui AY Hsu CJ Peng R Jiang N Xu XH. Global, regional prevalence, and risk factors of osteoporosis according to the World Health Organization diagnostic criteria: a systematic review and meta-analysis. Osteopor Int. (2022) 33:2137–53. doi: 10.1007/s00198-022-06454-3.PMID:35687123, PMID: 35687123

[12] Yuan S Larsson SC. Epidemiology of sarcopenia: prevalence, risk factors, and consequences. Metab Clin Exp. (2023) 144:155533. doi: 10.1016/j.metabol.2023.155533. PMID:36907247, PMID: 36907247

[13] Kanis JA Cooper C Rizzoli R Reginster JY. European guidance for the diagnosis and management of osteoporosis in postmenopausal women. Osteopor Int. (2019) 30:3–44. doi: 10.1007/s00198-018-4704-5, PMID: 30324412 PMC7026233

[14] McCloskey EV Harvey NC Johansson H Kanis JA. FRAX updates 2016. Curr Opin Rheumatol. (2016) 28:433–41. doi: 10.1097/bor.0000000000000304, PMID: 27163858

[15] Huang C-b Tan K Wu Z-y Yang L. Application of machine learning model to predict lacunar cerebral infarction in elderly patients with femoral neck fracture before surgery. BMC Geriatr. (2022) 22:912. doi: 10.1186/s12877-022-03631-1, PMID: 36443675 PMC9703654

[16] Clynes MA Gregson CL Bruyère O Cooper C Dennison EM. Osteosarcopenia: where osteoporosis and sarcopenia collide. Rheumatology. (2021) 60:529–37. doi: 10.1093/rheumatology/keaa755.PMID:33276373, PMID: 33276373

[17] Huang CB Hu JS Tan K Zhang W Xu TH Yang L. Application of machine learning model to predict osteoporosis based on abdominal computed tomography images of the psoas muscle: a retrospective study. BMC Geriatr. (2022) 22:796. doi: 10.1186/s12877-022-03502-9. PMID:36229793, PMID: 36229793 PMC9563158

[18] Kajiki Y Tsuji H Misawa H Nakahara R Tetsunaga T Yamane K. Psoas muscle index predicts osteoporosis and fracture risk in individuals with degenerative spinal disease. Nutrition. (2022) 93:111428. doi: 10.1016/j.nut.2021.111428. PMID:34474186, PMID: 34474186

[19] Murat S Dogruoz Karatekin B Demirdag F Kolbasi EN. Anthropometric and body composition measurements related to osteoporosis in geriatric population. Medeniyet Med J. (2021) 36:294–301. doi: 10.4274/MMJ.galenos.2021.32396. PMID:34937323, PMID: 34937323 PMC8694162

[20] Huang CB Lin DD Huang JQ Hu W. Based on CT at the third lumbar spine level, the skeletal muscle index and psoas muscle index can predict osteoporosis. BMC Musculoskelet Disord. (2022) 23:933. doi: 10.1186/s12891-022-05887-5. PMID:36280811, PMID: 36280811 PMC9590212

[21] Benedetto U Head SJ Angelini GD Blackstone EH. Statistical primer: propensity score matching and its alternatives. Eur J Cardiothor Surg. (2018) 53:1112–7. doi: 10.1093/ejcts/ezy167. PMID:29684154, PMID: 29684154

[22] Reiffel JA. Propensity score matching: the 'Devil is in the Details' where more may be hidden than you know. Am J Med. (2020) 133:178–81. doi: 10.1016/j.amjmed.2019.08.055. PMID:31618617, PMID: 31618617

[23] Lee WC Guntur AR Long F Rosen CJ. Energy metabolism of the osteoblast: implications for osteoporosis. Endocr Rev. (2017) 38:255–66. doi: 10.1210/er.2017-00064. PMID:28472361, PMID: 28472361 PMC5460680

[24] Li G Zhang L Wang D AIQudsy L Jiang JX Xu HY. Muscle-bone crosstalk and potential therapies for sarco-osteoporosis. J Cell Biochem. (2019) 120:14262–73. doi: 10.1002/jcb.28946, PMID: 31106446 PMC7331460

[25] Yakabe M Hosoi T Akishita M Ogawa S. Updated concept of sarcopenia based on muscle-bone relationship. J Bone Miner Metab. (2020) 38:7–13. doi: 10.1007/s00774-019-01048-2. PMID:31583540, PMID: 31583540

[26] Colaianni G Storlino G Sanesi L Colucci S Grano M. Myokines and Osteokines in the pathogenesis of muscle and bone diseases. Curr Osteoporos Rep. (2020) 18:401–7. doi: 10.1007/s11914-020-00600-8, PMID: 32514668

[27] Ito K Ookawara S Hibino Y Imai S Fueki M Bandai Y. Skeletal muscle mass index is positively associated with bone mineral density in hemodialysis patients. Front Med. (2020) 7:187. doi: 10.3389/fmed.2020.00187, PMID: 32478086 PMC7242614

[28] Huang M Hung VW Li TK Law SW Wang Y Chen S. Performance of HR-pQCT, DXA, and FRAX in the discrimination of asymptomatic vertebral fracture in postmenopausal Chinese women. Arch Osteoporos. (2021) 16:125. doi: 10.1007/s11657-021-00939-0. PMID:34480663, PMID: 34480663 PMC8418592

[29] Bougioukli S Κollia P Koromila T Varitimidis S Hantes M Karachalios T. Failure in diagnosis and under-treatment of osteoporosis in elderly patients with fragility fractures. J Bone Miner Metab. (2019) 37:327–35. doi: 10.1007/s00774-018-0923-2, PMID: 29667007

[30] Guzon-Illescas O Perez Fernandez E Crespí Villarias N Quirós Donate FJ Peña M Alonso-Blas C. Mortality after osteoporotic hip fracture: incidence, trends, and associated factors. J Orthop Surg Res. (2019) 14:203. doi: 10.1186/s13018-019-1226-6. PMID:31272470, PMID: 31272470 PMC6610901

[31] Matsumoto H Tanimura C Kushida D Osaka H Kawabata Y Hagino H. FRAX score and recent fall history predict the incidence for sarcopenia in community-dwelling older adults: a prospective cohort study. Osteoporos Int. (2020) 31:1985–94. doi: 10.1007/s00198-020-05447-4. PMID:32448948, PMID: 32448948

[32] Yu R Leung J Woo J. Sarcopenia combined with FRAX probabilities improves fracture risk prediction in older Chinese men. J Am Med Dir Assoc. (2014) 15:918–23. doi: 10.1016/j.jamda.2014.07.011. PMID:25262197, PMID: 25262197

[33] Pasco JA Mohebbi M Tembo MC Holloway-Kew KL Hyde NK Williams LJ. Repurposing a fracture risk calculator (FRAX) as a screening tool for women at risk for sarcopenia. Osteoporos Int. (2020) 31:1389–94. doi: 10.1007/s00198-020-05376-2. PMID:32185435, PMID: 32185435

[34] Siris ES Baim S Nattiv A. Primary care use of FRAX: absolute fracture risk assessment in postmenopausal women and older men. Postgrad Med. (2010) 122:82–90. doi: 10.3810/pgm.2010.01.2102. PMID:20107292, PMID: 20107292

